# Three-dimensional DNA nanostructures to improve the hyperbranched hybridization chain reaction†
†This work is dedicated to 100th anniversary of Nankai University.
‡
‡Electronic supplementary information (ESI) available. See DOI: 10.1039/c9sc02281c


**DOI:** 10.1039/c9sc02281c

**Published:** 2019-08-29

**Authors:** Jing Wang, Dong-Xia Wang, Jia-Yi Ma, Ya-Xin Wang, De-Ming Kong

**Affiliations:** a State Key Laboratory of Medicinal Chemical Biology , Tianjin Key Laboratory of Biosensing and Molecular Recognition , Research Centre for Analytical Sciences , College of Chemistry , Nankai University , Tianjin 300071 , P. R. China . Email: kongdem@nankai.edu.cn; b Collaborative Innovation Center of Chemical Science and Engineering (Tianjin) , Tianjin , 300071 , P. R. China

## Abstract

Nonenzymatic nucleic acid amplification techniques (*e.g.* the hybridization chain reaction, HCR) have shown promising potential for amplified detection of biomarkers. However, the traditional HCR occurs through random diffusion of DNA hairpins, making the kinetics and efficiency quite low. By assembling DNA hairpins at the vertexes of tetrahedral DNA nanostructures (TDNs), the reaction kinetics of the HCR is greatly accelerated due to the synergetic contributions of multiple reaction orientations, increased collision probability and enhanced local concentrations. The proposed quadrivalent TDN (qTDN)-mediated hyperbranched HCR has a ∼70-fold faster reaction rate than the traditional HCR. The approximately 76% fluorescence resonance energy transfer (FRET) efficiency obtained is the highest in the reported DNA-based FRET sensing systems as far as we know. Moreover, qTDNs modified by hairpins can easily load drugs, freely traverse plasma membranes and be rapidly cross-linked *via* the target-triggered HCR in live cells. The reduced freedom of movement as a result of the large crosslinked structure might constrain the hyperbranched HCR in a confined environment, thus making it a promising candidate for *in situ* imaging and photodynamic therapy. Hence, we present a paradigm of perfect integration of DNA nanotechnology with nucleic acid amplification, thus paving a promising way to the improved performance of nucleic acid amplification techniques and their wider application.

## Abstract 2

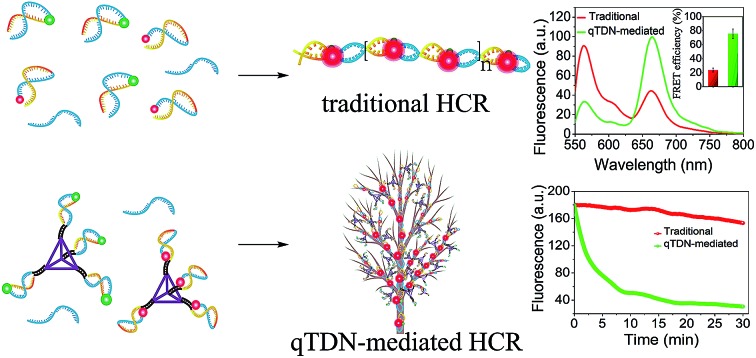
Nonenzymatic nucleic acid amplification techniques (*e.g.* the hybridization chain reaction, HCR) have shown promising potential for amplified detection of biomarkers.

## Introduction

Advances in genomics, proteomics and molecular pathology have generated many candidate biomarkers with potential clinical value.^1^ The detection of these biomarkers (such as proteins, nucleic acids, enzymes and small biomolecules) is of great significance for disease diagnosis, prognosis and treatment selection. Previous research has focused on *in vitro* detection and characterization of biomarkers. In order to clarify the important role of these biomarkers in the occurrence and development of disease and to achieve personalized therapy, increasing emphasis has been placed on rapid and sensitive imaging of the temporal and spatial distribution of biomarkers at the single-cell level. However, the intrinsic characteristics of most biomarkers, including low abundance and high structural similarity among family members, make their accurate detection challenging.

DNA-based biosensing and bioimaging studies have attracted more and more attention in recent decades.^2^ Versatile nucleic acid amplification techniques have been explored and successfully used to enhance the sensitivity of biosensors. For example, the polymerase chain reaction,^3^ rolling-circle amplification,^4^ the strand displacement reaction^5^ and duplex-specific nuclease^6^ have played important roles in the *in vitro* detection of low-abundance biomarkers with high sensitivity. These amplification methods require the involvement of multiple proteases. However, the delivery of proteases into live cells is currently difficult and may interfere with the expression levels of some biomarkers,^7^ which may not be suitable for live-cell nanoscale detection of intracellular targets. Therefore, some nonenzymatic amplification techniques, including the hybridization chain reaction (HCR)^8^–^13^ and catalytic hairpin assembly (CHA),^14^,^15^ are increasingly being used in intracellular assays. However, traditional nonenzymatic reactions have three inevitable shortcomings of relatively slow kinetics, low cell internalization efficiency and weak biostability of DNA probes.^15^ In recent years, some constructive work has been proposed to overcome the slow kinetics of nonenzymatic reactions by utilizing spatial constraint. Seelig *et al.* designed a type of localized molecular circuit on DNA origami for fast and efficient DNA computing, significantly accelerating the nonenzymatic amplification reactions.^16^ Also, Reif *et al.* developed a localized HCR on a DNA origami rectangle, in which the hairpins were successfully programmed to undergo a cascade HCR.^17^ Almost at the same time, Merkx *et al.* conducted DNA-based computing on a supramolecular polymer, and the kinetics of strand displacement and strand exchange reactions were accelerated 100-fold.^18^ Besides, some innovative work regarding proximity-induced hybridization has been proposed for dynamic visualization. Huang *et al.* designed a proximity-induced DNA assembly for visualization of protein-specific glycosylation and protein dimerization.^19^,^20^ All these attempts utilize spatial-confinement effects to accelerate reaction kinetics. However, under physiological conditions, the reactants are still in random mode in the bulk medium, resulting in low collision efficiency. According to the effective collision theory, the reaction rate can be effectively boosted by increasing concentrations as well as orientations of reactants.

Inspired by this theory, we developed a tetrahedral DNA nanostructure-mediated nonenzymatic nucleic acid amplification reaction (*e.g.* the HCR) to accelerate the reaction kinetics. By assembling DNA hairpins at the vertexes of quadrivalent tetrahedral DNA nanostructures (qTDNs), the reaction kinetics of the HCR is greatly accelerated due to the synergetic contributions of multiple reaction orientations, increased collision probability and enhanced local concentrations. Moreover, the participation of qTDNs can also efficiently improve the cell internalization efficiency and biostability of DNA probes. The qTDN-mediated HCR can solve the three major problems faced by the traditional HCR all at once. As a proof-of-concept, we demonstrated that it is a promising candidate for *in situ* imaging and photodynamic therapy (PDT).

## Results and discussion

### Design of the qTDN-mediated HCR

**Scheme 1:**
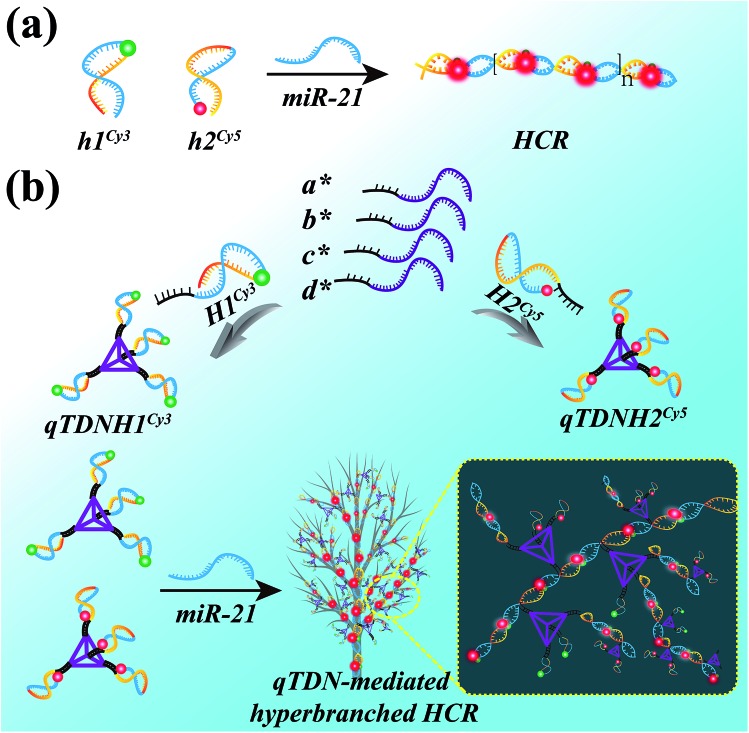
Schematic illustration of (a) the traditional HCR and (b) qTDN-mediated hyperbranched HCR.

The HCR is an isothermal enzyme-free process with the advantages of high simplicity and versatility.^8^ The traditional HCR uses two hairpin probes (h1 and h2, Table S1‡) to perform target-triggered alternative assembly, producing linear, long-stranded duplex products, resulting in fluorescence resonance energy transfer (FRET) signal output (Scheme 1a).

To accelerate the reaction kinetics of the HCR and promote its applications in live cells, we designed a qTDN-mediated HCR strategy (Scheme 1b). A TDN, the simplest DNA polyhedron nanostructure, is particularly suitable for constructing DNA nanostructures as building modules for its multivalency, high cellular uptake capacity and improved biostability.^21^–^24^ The hairpin probes, which are used in the traditional HCR, can be easily linked to the vertexes of qTDNs. By adding a C_2_A_22_ sequence to the 5′-end of each of the four oligonucleotides (a*, b*, c* and d*, Table S1‡), a qTDN with four C_2_A_22_ tails can be obtained. To achieve the assembly of hairpin probes on qTDN, we added a T_22_G_2_ tail to each of them (H1 and H2, Table S1‡). By virtue of the hybridization between C_2_A_22_ and T_22_G_2_, H1 and H2 could be separately modified on different TDNs, thus forming hairpin-containing qTDNs (*e.g.*, qTDNH1 and qTDNH2, in which four H1 and H2 molecules were assembled on one qTDN, respectively; Scheme 1b). Because the four vertexes in the qTDN are separated from each other, the high steric hindrance generated by a dense DNA nanostructure can be efficiently avoided and can ensure the formation of hyperbranched qTDN-assembled superstructures. When the proposed qTDN-mediated HCR is triggered by a specified target (*e.g.* microRNA), H1 in qTDNH1 and H2 in qTDNH2 will be alternately opened, and qTDN-assembled DNA superstructures will be formed. Each qTDN contains four hairpin probes. These hairpin probes locate in different orientations, which might increase the collision probability between two hairpins from different qTDNs. In addition, the three-dimensional structure of the qTDN enables the qTDN-mediated HCR to be performed towards different orientations, which is beneficial for grabbing free reactants from the surrounding environment. What's more, once the hairpin at one qTDN vertex participates in the HCR reaction, the other three are naturally brought into the DNA superstructures, resulting in a great increase of the local concentrations of reactants. The synergy of increased collision chance, multiple reaction directions and increased local reactant concentrations will certainly be helpful to achieve accelerated HCR reaction kinetics.

### Synthesis and characterization of DNA superstructures

**Fig. 1:**
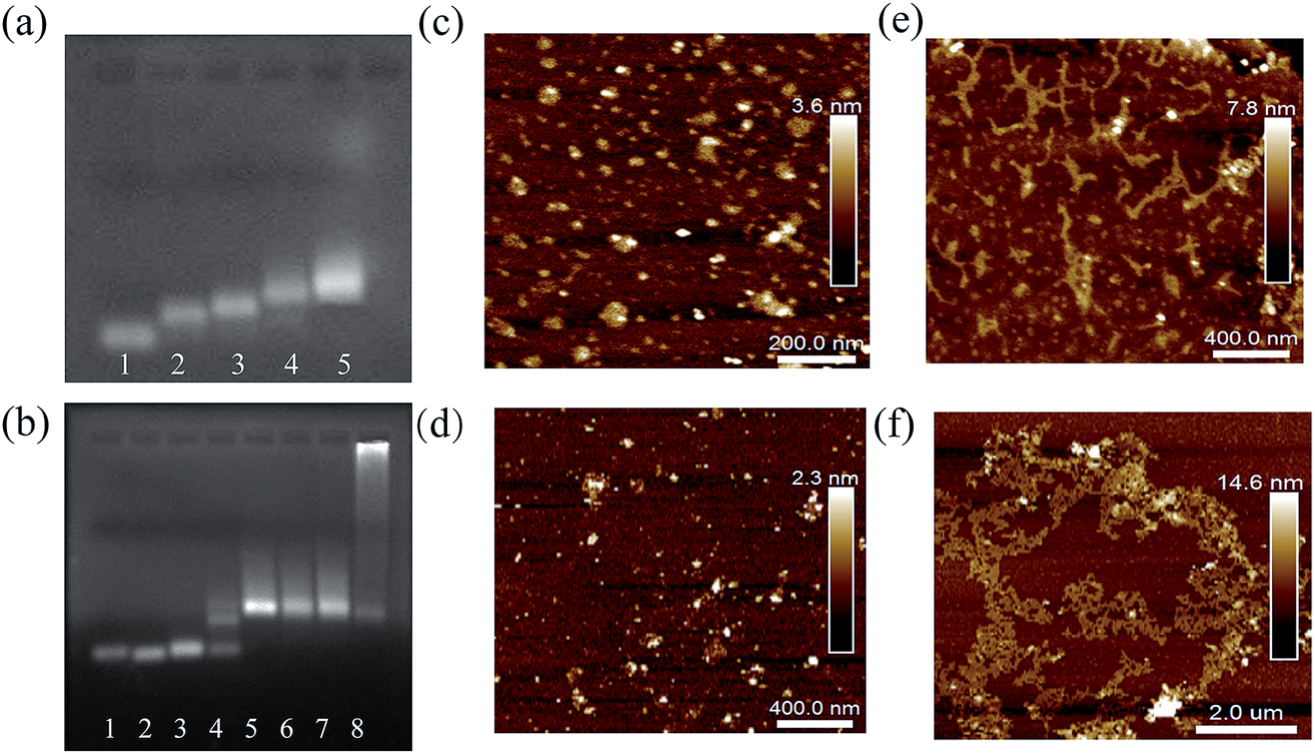
AGE and AFM characterization results of the traditional HCR and qTDN-mediated hyperbranched HCR. (a and b) Gel images showing (a) the successful construction of the qTDN and qTDNH1 (Lanes 1 → 5: a*; a* + b*; a* + b* + c*; a* + b* + c* + d*; a* + b* + c* + d* + H1) and (b) the successful performance of the miR-21-initiated traditional HCR or TDN-mediated hyperbranched HCR. Lanes 1 → 8: h1; h2; h1 + h2; h1 + h2 + miR-21; qTDNH1; qTDNH2; qTDNH1 + qTDNH2; qTDNH1 + qTDNH2 + miR-21. (c–f) AFM images of the morphology of (c) h1 + h2, (d) qTDNH1 + qTDNH2, (e) h1 + h2 + miR-21 and (f) qTDNH1 + qTDNH2 + miR-21 in air.

Several characterization methods were used to demonstrate the feasibility of the proposed qTDN-mediated hyperbranched HCR. First of all, agarose gel electrophoresis (AGE) was used to verify the preparation of qTDNs and qTDNH1. As shown in Fig. 1a, with the successive addition of oligonucleotides a*, b*, c*, d* and H1, a clear decrease in electrophoretic mobility was observed, suggesting that the qTDN and qTDNH1 were successfully prepared. Next, the initiator-triggered hyperbranched HCR was demonstrated. We chose miR-21, a microRNA overexpressed in many cancers,^25^,^26^ as the initiator to trigger the hyperbranched HCR between qTDNH1 and qTDNH2, and the results were compared with those of the traditional HCR between h1 and h2 (Fig. 1b), in which no qTDNs participated. Without miR-21, neither h1/h2 nor qTDNH1/qTDNH2 spontaneously assembled, thus indicating that all of these hairpins were sufficiently stable without false hybridization. In the presence of an initiator, a DNA superstructure with a greatly increased molecular mass was formed by the qTDN-mediated hyperbranched HCR (qTDNH1 + qTDNH2 + miR-21), as reflected by the appearance of a bright DNA band with very slow migration. In contrast, the traditional HCR (h1 + h2 + miR-21) yielded only DNA bands with a slightly increased molecular mass. Then, atomic force microscopy (AFM) was used to intuitively view the distinct HCR products. Without the initiator, the samples of h1/h2 and qTDNH1/qTDNH2 mixtures were randomly distributed in a monodisperse state with uniform sizes (Fig. 1c and d). In contrast, in the presence of miR-21, submicrometer-long dsDNA nanowires were observed for the h1/h2-based traditional HCR (Fig. 1e) and micrometer-size DNA superstructures were yielded by the qTDNH1/qTDNH2-based hyperbranched HCR (Fig. 1f). These results revealed that the proposed hyperbranched HCR could be performed smoothly after assembling metastable hairpins on qTDNs, producing DNA superstructures with large sizes.

### FRET measurement

Having demonstrated the feasibility of the qTDN-mediated hyperbranched HCR, we then verify its accelerated reaction kinetics compared to those of the traditional HCR using fluorescence assays. The traditional HCR can amplify a single molecular recognition event over one thousand-fold, thus making it a potential signal amplifier for detecting targets with low abundance. However, it has relatively slow kinetics inappropriate for rapid analysis. In addition, a prolonged reaction time may also increase the risk of undesired hybridization reactions.

**Fig. 2:**
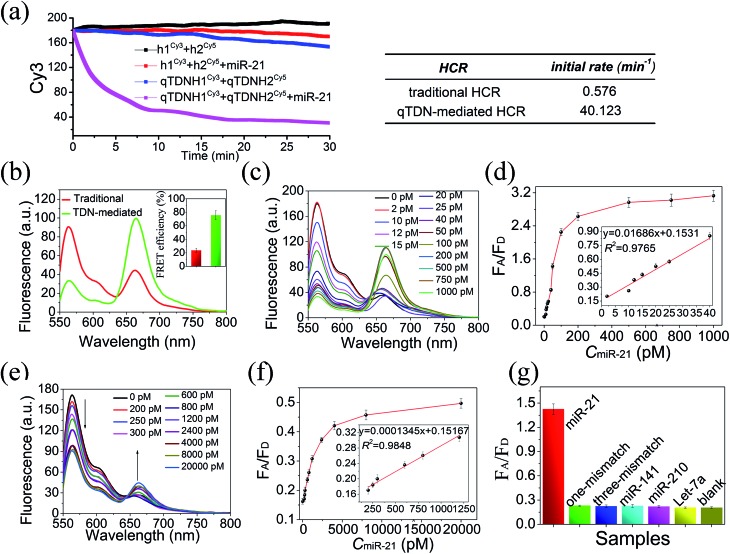
Reaction kinetics, FRET efficiency and miR-21-sensing applications of the qTDN-mediated hyperbranched HCR and traditional HCR. (a) Time-dependent Cy3 fluorescence changes (at 560 nm) of the systems in the absence or presence of miR-21. The initial rates of these two kinds of HCRs are outlined in the table below. The concentrations of hairpins of h1^Cy3^, h2^Cy5^, H1^Cy3^ and H2^Cy5^ were 50 nM and the concentration of qTDN in qTDNH1 or qTDNH2 was 12.5 nM. 20 nM miR-21 was added in the reaction buffer (20 mM Tris–HCl, 10 mM MgCl_2_, pH 7.4). (b) Fluorescence spectra and the calculated FRET efficiencies (inset) of the two systems (*λ*_ex_ = 540 nm). (c and d) qTDN-mediated hyperbranched HCR-based miR-21-sensing. (c) Fluorescence spectral changes of the sensing system in the presence of different concentrations of miR-21. (d) miR-21 concentration-dependent changes in *F*_A_/*F*_D_ and the linear relationship (inset) between *F*_A_/*F*_D_ and the miR-21 concentration in the range of 2–40 pM (*F*_A_ and *F*_D_ are the fluorescence of Cy5 at 660 nm and of Cy3 at 560 nm, respectively). (e and f) Traditional HCR-based miR-21-sensing. (e) Fluorescence spectral changes of the sensing system in the presence of different concentrations of miR-21. (d) miR-21 concentration-dependent changes in *F*_A_/*F*_D_ and the linear relationship (inset) between *F*_A_/*F*_D_ and the miR-21 concentration in the range of 200–1200 pM. (g) Selectivity of the qTDN-mediated hyperbranched HCR-based miR-21-sensing system. 50 pM miR-21 or a 20 nM concentration of other different microRNAs was added.

To achieve real-time monitoring of the HCR kinetics, we labeled h1 and H1 with the fluorescence donor Cy3 and h2 and H2 with the fluorescence acceptor Cy5, respectively. When the traditional and qTDN-mediated HCRs were successfully triggered by miR-21, the FRET between them shifted from an “off” to an “on” state. This signal output mode is superior to fluorophore/quencher systems, because false positive signals caused by probe degradation can be efficiently prevented.^27^,^28^ By recording the fluorescence decrease of Cy3 with reaction time, it showed that the initial reaction rate of the qTDN-mediated hyperbranched HCR was about 70-fold faster than that of the traditional HCR (Fig. 2a), confirming the vital role of qTDNs in the significant improvement of HCR reaction kinetics. Only 20 min was needed for the qTDN-mediated hyperbranched HCR-based sensing system to achieve a stable signal output under the given conditions. More interestingly, when miR-21 was added in equal concentrations, the FRET efficiency of the hyperbranched HCR reached approximately 76%, 3.2-fold higher than that of the traditional HCR (Fig. 2b). This is because in the DNA superstructures formed by the qTDN-mediated hyperbranched HCR, one Cy5 fluorophore will be in close contact with several Cy3 fluorophores in an effective FRET distance range and *vice versa*. Such an extraordinarily high FRET efficiency might make the qTDN-mediated hyperbranched HCR superior in biosensing and bioimaging applications.

Then, the *in vitro* applications of the qTDN-mediated hyperbranched HCR and traditional HCR in target miR-21-sensing were compared. As shown in Fig. 2c–f, the limit of detection (LOD) of the qTDN-mediated hyperbranched HCR (2.14 pM) was much better than that of the traditional HCR (207 pM) on the basis of the 3*σ*/*S* rule (*σ* is represents the standard deviation of the blank control (*N* = 10) and *S* is the slope of the calibration curve). Besides, such an excellent specificity of the hyperbranched HCR was achieved that even one-base mismatched target microRNA did not yield an observable FRET signal change, as compared with the blank control (Fig. 2g). The risk reduction of undesired hybridization reactions might be attributed to the accelerated kinetics and shortened reaction time.

### TDN-mediated HCR using differently “valent” TDN-assembled hairpins

To further elucidate the contribution of increased reaction orientations and local concentrations to the accelerated reaction kinetics of the HCR, different valent TDN-assembled hairpins were prepared to perform the TDN-mediated HCR. Since the TDN has four vertexes, a number of hairpins, ranging from one to four, can be easily modified on the TDN with varied valence. Herein, the valence of the TDN is defined as the number of C_2_A_22_ tails added at the TDN vertexes. For example, a bivalent TDN, prepared by hybridization of four oligonucleotides (a*, b*, c and d, Table S1‡), has two C_2_A_22_ tails. Using this bivalent TDN, a bTDN-assembled HCR reactant with two hairpin units (bTDNH1 or bTDNH2) can be obtained. In the above experiment, qTDN-assembled HCR reactants (qTDNH1 and qTDNH2) were used to demonstrate the feasibility of the qTDN-mediated hyperbranched HCR. Next, other valent TDN-assembled HCR reactants were prepared and used for TDN-mediated HCR studies. Their detailed compositions and integral structures are listed in Table S2 and Fig. S1a and b.‡ AGE characterization (Fig. S1c‡) demonstrated that differently valent TDN-assembled hairpins could successfully be prepared and all of them could perform the initiator miR-21-triggered HCR. The molecular weight of the HCR products notably increased with the valence of the TDN, and bulk DNA superstructures were formed only when the TDN valence was increased to three. Hence, AFM in air was utilized for a more direct visualization of TDN-mediated HCR reactions. It was clearly shown that TDN-mediated HCR reactions could be successfully triggered by miR-21, and HCR products with increasing sizes were observed with an increase in the TDN valence (Fig. S1c–j‡). DNA superstructures in the micrometer size range could be yielded by trivalent and quadrivalent TDNHs, in agreement with the AGE results. Although the results of AFM imaging in air showed a collapsed appearance due to DNA dehydration and strong electrostatic interactions between the DNA and mica substrate surface, the widest and highest amounts of HCR products were clearly yielded by the qTDN (qTDNH1/qTDNH2), thus indicating that higher valences can yield more stretchable and steric structures.

Then, the reaction kinetics of the different valence TDN-mediated HCR reaction was compared. By recording the Cy3 fluorescence decrease with the reaction time (Fig. S2a‡), we found that the HCR kinetics was highly dependent on the TDN valence. The monovalent TDN-mediated HCR showed similar reaction kinetics to the traditional HCR. Yet, the qTDN-mediated HCR exhibited faster kinetics, its Cy3 fluorescence nearly reached a plateau in 20 min. A valence-dependent increase of the reaction kinetics perfectly demonstrated our proposed mechanism, namely, accelerated reaction kinetics achieved by increasing the reaction orientations and local reactant concentrations. Owing to the steric and stretchable structures of high valent TDNs, the HCR can be simultaneously triggered in several directions, thus making crosslinking of TDN-assembled hairpins possible. Once a vertex of a TDN participates in the HCR, the other three vertexes are naturally brought into the assembly, thus greatly increasing the local reactant (hairpin) concentrations and speeding up subsequent HCRs. Herein, we demonstrated that the reaction kinetics of DNA assembly can be accelerated by using multivalent DNA nanostructures, thereby proposing a new paradigm of accelerating nonenzymatic nucleic acid amplification reactions.

The FRET efficiencies of sensing systems were also greatly increased with a valence from one to three, and a comparable one resulted from tri- and quadri-valence (Fig. S2b‡). This can be explained by the hyperbranched DNA superstructures (Fig. S1‡). In a relatively compact superstructure, one Cy3 fluorophore might come into contact with multiple Cy5 fluorophores in an effective FRET distance range and *vice versa*. Approximately 76% FRET efficiency achieved by the qTDN-mediated hyperbranched HCR is also the highest value for reported DNA-based FRET sensing systems.^29^–^33^

### Intracellular target imaging *via* the qTDN-mediated HCR

**Fig. 3:**
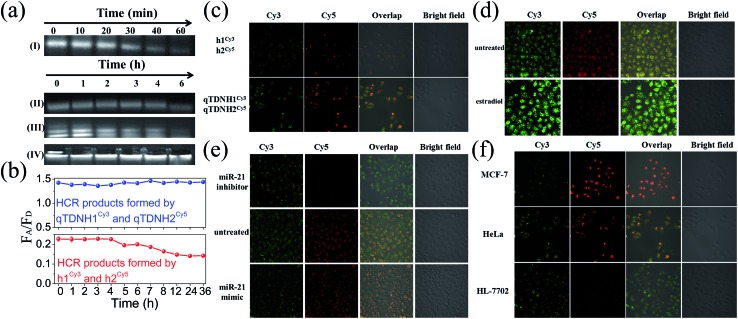
*In situ* miR-21 imaging *via* the qTDN-mediated HCR in live cells. Biostability, biocompatibility and confocal laser scanning microscopy (CLSM) images. (a and b) Biostability characterization of hairpins and HCR products. The concentrations of hairpins h1 and H1 were 1 μM and the concentration of qTDN was 0.25 μM. (a) AGE images of (I) h1, (II) qTDNH1, (III) HCR products formed by h1 and h2, and (IV) HCR products formed by qTDNH1 and qTDNH2 after treatment with 0.5 U mL^–1^ DNase I for different times; (b) FRET signal changes of HCR products formed by h1^Cy3^/h2^Cy5^ or qTDNH1^Cy3^/qTDNH2^Cy5^ after incubation with 10% fetal bovine serum for different times. (c) FRET images of miR-21 in HeLa cells after incubation with h1^Cy3^/h2^Cy5^ or qTDNH1^Cy3^/qTDNH2^Cy5^ for 4 h. (d) FRET images of miR-21 in untreated HeLa cells and in estradiol-treated HeLa cells. HeLa cells were treated with 10 nM estradiol for 24 h at 37 °C. (e) FRET images of miR-21 in untreated HeLa cells and miR-21 mimic- and inhibitor-transfected HeLa cells. (f) FRET images of miR-21 in MCF-7, HeLa and HL-7702 cells. In (c)–(f), the concentrations of hairpins of h1^Cy3^, h2^Cy5^, H1^Cy3^ and H2^Cy5^ were 50 nM and the concentration of qTDN in qTDNH1^Cy3^ or qTDNH2^Cy5^ was 12.5 nM.

Excellent biological stability and cell membrane penetrability are necessary for live cell applications. The intracellular applications of the traditional HCR are limited by the poor cell membrane penetrability and weak biostability of DNA hairpins. These drawbacks can be overcome by assembling hairpins on TDNs.^34^ DNA nanostructures, including TDNs, were reported to have much better cell internalization efficiencies than simple DNA strands *via* macropinocytosis and caveolae-mediated endocytosis pathways. It is reasonable to infer that hairpins can be easily and efficiently delivered into cells with the help of TDNs. Moreover, owing to the protection of TDNs, both qTDN-assembled hairpins and the corresponding HCR products showed much better stability than unprotected ones in the traditional HCR. AGE analysis (Fig. 3a) showed that the bands of qTDN-assembled hairpins (*e.g.* qTDNH1) were still clearly observed within 4 h after incubation with 0.5 U mL^–1^ DNase I, a powerful endonuclease capable of effectively degrading both single- and double-stranded DNAs. Hyperbranched HCR products formed by the qTDN-mediated HCR remained stable even after 6 h. Fluorescence analysis showed that after treatment in a cell culture medium, the traditional HCR products formed by h1^Cy3^ and h2^Cy5^ exhibited a time-dependent decrease in the FRET signal after 4 h, but the FRET signal of HCR products formed by qTDNH1^Cy3^ and qTDNH2^Cy5^ remained almost unchanged within 36 h (Fig. 3b). These results strongly demonstrated that the introduction of qTDNs endows both probes and HCR products with greatly improved stability. The enhanced biostability, as well as a FRET-based signal output strategy, efficiently prevented false-negative and false-positive results. In addition, fast reaction kinetics and reduced freedom of movement as a result of the large crosslinked framework might constrain HCR products in a confined environment, thus making the qTDN-mediated HCR a promising candidate for many applications, such as *in situ* imaging of targets in live cells.^35^

We next investigated the feasibility of *in situ* imaging of tumor-related microRNA in live cells. After incubation of a h1^Cy3^/h2^Cy5^ or qTDNH1^Cy3^/qTDNH2^Cy5^ mixture with HeLa cells (a human cervical cancer cell line known to overexpress miR-21 (ref. 24)), we found that fluorescence signals of qTDNH1^Cy3^/qTDNH2^Cy5^ were 1.6-fold higher than those of h1^Cy3^/h2^Cy5^ (Fig. 3c). In contrast, the FRET signals (ratio Cy5/Cy3) of both were almost the same, a little different from the solution results. These results suggested that qTDN-assembled hairpins were rapidly internalized into live cells without the help of transfection agents (Fig. S3‡) and subsequently DNA superstructure assembly occurs *via* the miR-21-triggered HCR. The observed FRET signals were highly miR-21 expression-dependent. Treatment of HeLa cells with estradiol, which downregulates miR-21 expression,^36^ greatly decreased the FRET signals (Fig. 3d). After transfection with miR-21 mimics or inhibitors, the HeLa cells showed clearly higher or lower FRET signals than the untreated control (Fig. 3e). To further demonstrate the specificity of microRNA detection in different live cells, we compared the FRET images of three kinds of cells with different miR-21 expression levels. As shown in Fig. 3f, MCF-7 cells (a breast cancer cell line) showed brighter FRET signals than HeLa cells, and the lowest FRET signals were observed in HL-7702 cells (a normal human epithelioid cell line). These results are consistent with the reported miR-21 expression levels in these cells,^37^ thus confirming that this method is capable of imaging low amounts of miR-21 in live cells and discriminating among different cells with different microRNA expression. Collectively, increased cell internalization, improved biostability, accelerated reaction kinetics, and extraordinarily high FRET efficiency make the qTDN-mediated HCR more suitable for applications in live cells than the traditional HCR.

### Universality of the proposed qTDN-mediated HCR

To demonstrate the universality of the proposed strategy, its feasibility for the detection of another microRNA (*e.g.*, Let-7a) was investigated. To achieve this, two fluorescent-labelled hairpins (H3^Cy3^ and H4^Cy5^, Table S1‡) that can perform a Let-7a-triggered HCR were assembled on the qTDN to construct another two qTDN-assembled hairpins qTDNH3^Cy3^ and qTDNH4^Cy5^. As shown in Fig. S4,‡ in the presence of target Let-7a, the qTDN-mediated hyperbranched HCR was successfully initiated, resulting in the decrease of Cy3 fluorescence and increase of Cy5 fluorescence and thus the increase of the FRET signal. Using such a qTDN-mediated hyperbranched HCR strategy, highly sensitive and specific detection of Let-7a with an LOD of 9.93 pM could be performed. After incubation of the qTDNH3^Cy3^/qTDNH4^Cy5^ mixture with MCF-7 cells (in which target Let-7a is expressed) for 4 h, obvious FRET signal output could be observed. When MCF-7 cells were transfected with a Let-7a mimic, a much higher FRET signal was observed compared to that of the untreated control. In contrast, Let-7a inhibitor-transfected cells gave a greatly decreased FRET signal output. All of these results suggested that the observed FRET signal was highly Let-7a expression-dependent, further suggesting that the proposed qTDN-mediated HCR method can be easily extended to the sensitive and reliable analysis of other target microRNAs in a biological matrix.

### Imaging-guided PDT treatment of cancer cells

**Fig. 4:**
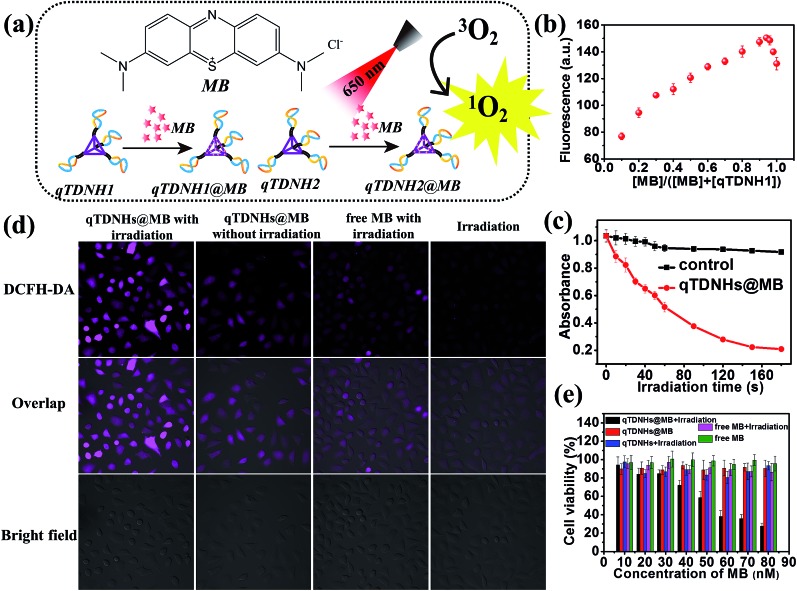
qTDN-assembled hairpin as a drug carrier of MB for PDT treatment of cancer cells. (a) Chemical structure of MB and its delivery by qTDNH1 and qTDNH2. (b) Job plot analysis of the interaction between MB and qTDNH1. (c) Time-dependent changes in the DPBF absorbance at 410 nm in the absence or presence of qTDNHs@MB (qTDNH1@MB + qTDNH2@MB) under 650 nm irradiation. (d) ^1^O_2_ generation in HeLa cells treated with different modes. (e) Viabilities of HeLa cells treated with different modes. “Irradiation” means treatment of cells with 650 nm light irradiation for 3 min.

The above experiments demonstrate that our proposed qTDN-mediated hyperbranched HCR works well for specific target-imaging detection in live cells, thus showing great potential in diagnostic applications. Because of their excellent stability, good cell internalization capacity and dense DNA nanostructure, TDNs are also promising carriers for the delivery of drugs that can intercalate into DNA duplexes (*e.g.* doxorubicin (Dox) and methylene blue (MB)). To widen the application range of the qTDN-mediated hyperbranched HCR in live cells, we next combined the dual functions of qTDN-assembled hairpins in imaging and drug delivery, and investigated the feasibility of the qTDN-mediated hyperbranched HCR in imaging-guided tumor-targeted PDT applications. As a proof-of-concept example, we used qTDNs as the delivery carrier of MB, the photosensitizer commonly used in tumor PDT (Fig. 4a).^38^,^39^ We first demonstrated that qTDN-assembled hairpins, including qTDNH1^Cy3^ and qTDNH2^Cy5^, have negligible cytotoxicity and excellent biocompatibility in live cells (Fig. S5‡). More than 90% viability was maintained by cells after treatment with a qTDNH1^Cy3^/qTDNH2^Cy5^ mixture for 24 h. And then we demonstrated that qTDN-assembled hairpins had high drug loading capability, and approximately 16 ± 2 MB molecules were calculated to be loaded per qTDNH1 or qTDNH2 (Fig. 4b). After being loaded in qTDNH1 and qTDNH2, MB maintained its singlet oxygen (^1^O_2_) generation ability after 650 nm irradiation, thus resulting in an irradiation time-dependent decrease in the absorbance of 1,3-diphenylisobenzofuran (DPBF), an extracellular ^1^O_2_ probe. In contrast, without irradiation, no obvious DPBF absorbance changes were observed (Fig. 4c). *In situ* intracellular generation of ^1^O_2_ was visually detected by using 2′,7′-dichlorodihydrofluorescein diacetate (DCFH-DA) as the fluorescent probe of ^1^O_2_ (Fig. 4d).^40^ HeLa cells treated with qTDNHs@MB (qTDNH1@MB and qTDNH2@MB) plus light irradiation showed much stronger fluorescence than the control cells treated with simple light irradiation or qTDNHs@MB in the dark, or with free MB plus light irradiation, thus confirming light-stimulated ^1^O_2_ generation by qTDNHs@MB in cells. It is also demonstrated that qTDNs are appropriate carriers that efficiently deliver hydrophilic MB with poor cell/tissue penetration into live cells.^41^,^42^ After irradiation with 650 nm light for 3 min, less than 30% of the cancer cells were alive when treated with qTDNHs@MB. Such a treatment showed markedly less cell viability than did the control groups (Fig. 4e), including those treated with qTDNHs@MB or free MB in the dark, and those treated with qTDNHs or free MB plus light irradiation, thus confirming the considerable PDT effect of qTDNHs@MB. The high viability (more than 90%) of HeLa cells treated with qTDNHs@MB in the dark indicated the negligible cytotoxicity of qTDNHs@MB. These results confirmed the feasibility of using MB-loaded qTDN-assembled hairpins in PDT treatment of cancer cells.

**Fig. 5:**
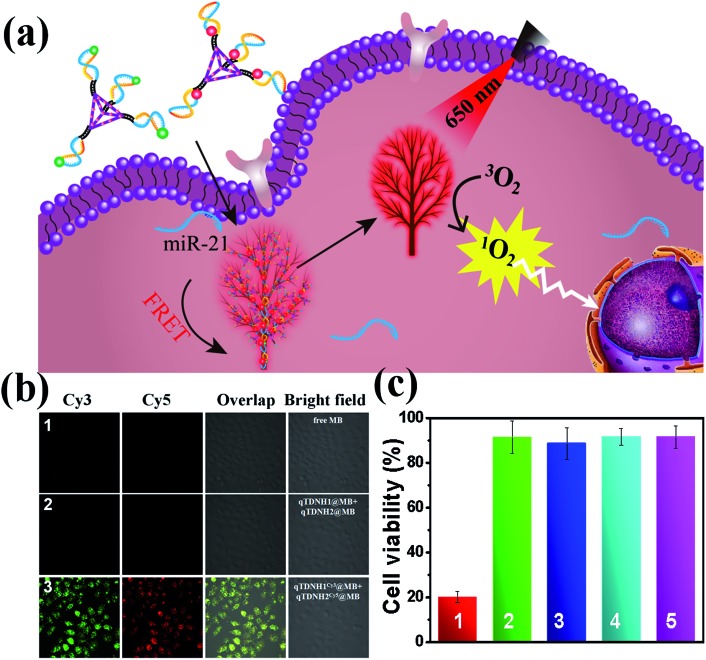
FRET imaging-guided PDT therapy using qTDNH1 and qTDNH2 as delivery carriers of MB. (a) Working mechanism. (b) Fluorescence images of HeLa cells treated by (1) free MB; (2) qTDNH1@MB + qTDNH2@MB; (3) qTDNH1^Cy3^@MB + qTDNH2^Cy5^@MB. (c) Cell viability of HeLa cells treated with (1) qTDNH1^Cy3^@MB + qTDNH2^Cy5^@MB plus irradiation; (2) qTDNH1^Cy3^@MB + qTDNH2^Cy5^@MB in the dark; (3) qTDNH1^Cy3^ + qTDNH2^Cy5^ plus irradiation; (4) free MB plus irradiation; (5) free MB in the dark. The concentration of each qTDN is 12.5 nM, that of H1 or H2 is 50 nM and that of MB is 80 nM.

Because both the absorption and emission spectra of MB overlap with those of Cy5 (Fig. S6b‡), we investigated whether maximum loading of MB in qTDNH1 and qTDNH2 ([MB] : [qTDNH1] (or [MB] : [qTDNH2]) = 16 : 1) might interfere with the FRET probing of microRNA by the qTDN-mediated hyperbranched HCR. Fortunately, although the FRET signal intensity partially decreased after maximum loading of MB in qTDNH1^Cy3^ and qTDNH2^Cy5^, sensitive detection of miR-21 was still achieved, and the LOD (3.83 pM, Fig. S6c–e‡) was still much better than those of the existing microRNA imaging methods (Table S3‡). *In situ* imaging of miR-21 in live cells provided a further demonstration. As shown in Fig. 5b, there was negligible MB fluorescence in cells incubated with free MB or qTDNH1@MB + qTDNH2@MB under Cy3 channel excitation, whereas strong FRET fluorescence was observed in the cells treated with qTDNH1^Cy3^@MB + qTDNH2^Cy5^@MB. After further treatment of qTDNH1^Cy3^@MB + qTDNH2^Cy5^@MB in cells with 650 nm irradiation for 3 min, the cell viability sharply decreased to ∼20%. Collectively, these results demonstrated that our strategy can work well in “sense and treat” assays, thus suggesting promising applications in activatable theranostics of diseases.

## Conclusions

In summary, we showed a new paradigm of a qTDN-mediated hyperbranched HCR strategy to accelerate the nonenzymatic HCR and promote its biological application in live cells. In this strategy, DNA hairpins, assembled at the vertexes of qTDNs, are used to perform the target-triggered hyperbranched HCR. The steric and multivalent characteristics of the qTDN can increase the collision probability of DNA hairpins by increasing the local concentrations and simultaneously initiating from multiple directions. The reaction rate of the qTDN-mediated hyperbranched HCR was about 70-fold faster than that of the traditional HCR. Due to the formation of hyperbranched HCR products, a stable FRET signal can be achieved within 20 min and up to 76% FRET efficiency could be obtained. The rapid reaction kinetics and ultrahigh FRET signal demonstrated its great potential in biosensing and bioimaging applications. In addition, the mediation of the qTDN can also promote the cell internalization of DNA hairpins, increase their biostability and thus facilitate the applications of the HCR in live cells. As a proof-of-concept, we demonstrated that the proposed qTDN-mediated hyperbranched HCR could be successfully applied in highly sensitive, specific and *in situ* imaging of miR-21 inside live cells. Furthermore, stable qTDNs can also serve as good carriers for some drugs, and we demonstrated that the loading of the photosensitizer MB did not obviously affect their imaging diagnosis capability; therefore, constructing a theranostic platform to perform imaging-guided disease therapy is possible. Above all, we presented a facile way to improve the performance of the nonenzymatic HCR by utilizing multivalent qTDNs. With the rapid development of DNA nanotechnology, high-order, multifunctional DNA superstructures can conceivably be assembled by using other precisely designed DNA nanostructures with more valence states as building modules, such as polyhedra, bipyramids, cubes or prisms. They provide a potential target-triggered, *in situ* assembly tool for further exploration in nanotechnology and biomedical fields.

## Conflicts of interest

There are no conflicts to declare.

## Supplementary Material

Supplementary information
