# Supplementary material for: Three-dimensional DNA nanostructures to improve the hyperbranched hybridization chain reaction
Source: Chem Sci. 2019 Aug 29;10(42):9758–67. doi: 10.1039/c9sc02281c (PMC6993746; doi:10.1039/c9sc02281c)
Supplement: Supplementary file 1 [file SC-010-C9SC02281C-s001.pdf]

Electronic Supplementary Material (ESI) for Chemical Science.
This journal is © The Royal Society of Chemistry 2019

SUPPLEMENTARY DATA

Three-dimensional DNA nanostructure to improve hyperbranched hybridization chain reaction

Jing Wang,[a] Dong-Xia Wang,[a] Jia-Yi Ma,[a] Ya-Xin Wang[a] and De-Ming Kong[a,b]

[a]State Key Laboratory of Medicinal Chemical Biology, Tianjin Key Laboratory of Biosensing and Molecular Recognition, Research Centre for Analytical Sciences, College of Chemistry, Nankai University, Tianjin 300071, P. R. China

[b]Collaborative Innovation Center of Chemical Science and Engineering (Tianjin), Tianjin, 300071, P. R. China

E-mail: kongdem@nankai.edu.cn

This work is dedicated to 100th anniversary of Nankai University

Contents

1. Experimental Section 3

1.1. Materials and instruments 3

1.2. Oligonucleotides used in this work 3

1.3. Modules and assemblies 5

1.4. Experimental section 5

2. AGE and AFM characterization of TDN-assembled hairpins and their HCR products 8

3. Reaction kinetics and FRET efficiency of different valence TDN-mediated HCR 9

4. FRET images of miR-21 in HeLa cells at different time points 10

5. FRET measurement of Let-7a in solutions and MCF-7 cells 11

6. Cytotoxicities of hairpins and qTDN-assembled hairpins 12

7. Effects of MB loading on FRET-based miR-21-sensing 13

8. Comparation of our microRNA-sensing system with reported ones 14

References 15

1. Experimental Section

1.1. Materials and instruments

All DNA and RNA oligonucleotides (Table S1) were purchased from Sangon Biotech Co., Ltd. (Shanghai, China) and purified by high-performance liquid chromatography. 1,3-Diphenylisobenzofuran (DPBF), 2',7'-dichlorodihydrofluorescein diacetate (DCFH-DA) and estradiol were obtained from Sigma-Aldrich. Lipofectamine 3000 transfection reagent was purchased from Invitrogen (USA). Methylene blue (MB) was purchased from Acros Organics. Deoxyribonucleases I (DNase I) was obtained from New England Biolabs Beijing Ltd. All reagents used in this work were of analytical grade and directly used without further purification. Deionized and sterilized water (resistance >18 MΩ·cm) was used throughout the experiments.

UV spectral measurements were performed on an Agilent Technologies Cary 60 UV/Vis spectrometer. All fluorescence measurements were carried out on a Shimadzu RF-5301 PC fluorescence spectrometer (Shimadzu Ltd., Japan). AFM characterization was observed by Bruker Multimode V8 Scanning Probe Microscopy (USA). The images of cells were visualized on an inverted confocal microscope (A1R Confocal System, Nikon).

1.2. Oligonucleotides used in this work

Table S1. Sequences of all oligonucleotides used in this work

Oligonucleotides	sequence (5'-3')
a	ACATTCCTAAGTCTGAAACATTACAGCTTGCTACACGAGAAGAGCCGCCATAGTA
b	TATCACCAGGCAGTTGACAGTGTAGCAAGCTGTAATAGATGCGAGGGTCCAATAC
c	TCAACTGCCTGGTGATAAAACGACACTACGTGGGAATCTACTATGGCGGCTCTTC
d	TTCAGACTTAGGAATGTGCTTCCCACGTAGTGTCGTTTGTATTGGACCCTCGCAT
a*	CCAAAAAAAAAAAAAAAAAAAAAAACATTCCTAAGTCTGAAACATTACAGCTTGCTACA CGAGAAGAGCCGCCATAGTA
b*	CCAAAAAAAAAAAAAAAAAAAAAAATATCACCAGGCAGTTGACAGTGTAGCAAGCTGTA ATAGATGCGAGGGTCCAATAC
c*	CCAAAAAAAAAAAAAAAAAAAAAAATCAACTGCCTGGTGATAAAACGACACTACGTGGG AATCTACTATGGCGGCTCTTC
d*	CCAAAAAAAAAAAAAAAAAAAAAAATTCAGACTTAGGAATGTGCTTCCCACGTAGTGTCG TTTGTATTGGACCCTCGCAT
h1	TCAACATCAGTCTGATAAGCTACTAAGTTAGCTTATCAGACTG

h2	TAGCTTATCAGACTGATGTTGACAGTCTGATAAGCTAACTTAG
h1^{Cy3}	TCAACATCAGTCTGATAAGCTAC/iCy3dT/AAGTTAGCTTATCAGACTG
h2^{Cy5}	TAGCTTATCAGACTGATGTTGACAGTCTGATAAGCTAACTTAG-Cy5
H1	TTTTTTTTTTTTTTTTTTTTTTGGTCAACATCAGTCTGATAAGCTACTAAGTTAGCTTATCAGACTG
H2	TTTTTTTTTTTTTTTTTTTTTTGGTAGCTTATCAGACTGATGTTGACAGTCTGATAAGCTAACTTAG
H1^{Cy3}	TTTTTTTTTTTTTTTTTTTTTTGGTCAACATCAGTCTGATAAGCTAC/iCy3dT/AAGTTAGCTTATCAGACTG
H2^{Cy5}	TTTTTTTTTTTTTTTTTTTTTTGGTAGCTTATCAGACTGATGTTGACAGTCTGATAAGCTAACTTAG-Cy5
H3^{Cy3}	TTTTTTTTTTTTTTTTTTTTTTGGAGTAGGTTGTATAGTTCAAAGTAACTATACAACCTACTACCTCA-Cy3
H4^{Cy5}	TTTTTTTTTTTTTTTTTTTTTTGGACTTTGAACTATACAACCTACT/iCy5dT/GAGGTAGTAGGTTGTATAGTT
miR-21	UAGCUUAUCAGACUGAUGUUGA
One-mismatchmiR-21	UAGGUUAUCAGACUGAUGUUGA
Three-mismatchmiR-21	UAGCUAAUCAGACCGAUGUAGA
miR-21 mimic	TAGCTTATCAGACTGATGTTGA
miR-21 inhibitor	TCAACATCAGTCTGATAAGCTA
Let-7a	UGAGGUAGUAGGUUGUAUAGUU
Let-7a mimic	TGAGGTAGTAGGTTGTATAGTT
Let-7a inhibitor	AACTATACAACCTACTACCTCA
miR-210	CUGUGCGUGUGACAGCGGCUGA
miR-141	UAACACUGUCUGGUAAAGAUGG

Modules and assemblies

Table S2. Preparation of TDN-assembled hairpins with different valences

	TDN-assembled haiprin		Fluorescent labelled TDN-assembled haiprin	
Zerovalent	zTDN	abcd=1: 1: 1: 1		
Monovalent	mTDNH1	a*bcdH1=1: 1: 1: 1: 1	mTDNH1^{Cy3}	a*bcdH1^{Cy3}=1: 1: 1: 1: 1
	mTDNH2	a*bcdH2=1: 1: 1: 1: 1	mTDNH2^{Cy5}	a*bcdH2^{Cy5}=1: 1: 1: 1: 1
Bivalent	bTDNH1	a*b*cdH1=1: 1: 1: 1: 2	bTDNH1^{Cy3}	a*b*cdH1^{Cy3} =1: 1: 1: 1: 2
	bTDNH2	a*b*cdH2=1: 1: 1: 1: 2	bTDNH2^{Cy5}	a*b*cdH2^{Cy5}=1: 1: 1: 1: 2
Trivalent	tTDNH1	a*b*c*dH1=1: 1: 1: 1: 3	tTDNH1^{Cy3}	a*b*c*dH1^{Cy3}=1: 1: 1: 1: 3
	tTDNH2	a*b*c*dH2=1: 1: 1: 1: 3	tTDNH2^{Cy5}	a*b*c*dH2^{Cy5}=1: 1: 1: 1: 3
Quadrivalent	qTDNH1	a*b*c*d*H1=1: 1: 1: 1: 4	qTDNH1^{Cy3}	a*b*c*d*H1^{Cy3}=1: 1: 1: 1: 4
	qTDNH2	a*b*c*d*H2=1: 1: 1: 1: 4	qTDNH2^{Cy5}	a*b*c*d*H2^{Cy5}=1: 1: 1: 1: 4
TDN	referred to all kinds of tetrahedrons from zerovalent to quadrivalent			

1.4. Experimental section

Construction of TDN-assembled hairpins

TDNs with different valences were prepared by mixing corresponding oligonucleotide strands (Table S1) in TM buffer (20 mM Tris-HCl, 50 mM $MgCl_2$, pH = 8.0). Unless otherwise stated, the concentration of a or a* is 1 µM and H1, H2 changes with different valences. For instance, the mixtures of qTDNH1 (a*, b*, c*, d* are 1 µM and H1 is 4 µM) were heated at 95 °C for 5 min and then cooled to 4 °C within 1 min. The obtained modules were then directly used for characterization without further fractionation or purification. To achieve the loading of MB in modules, MB was mixed with the prepared modules (*e.g.* qTDNH1 or qTDNH2) at 37 °C for 1 h and then purified by ultrafiltration (30 kDa molecular weight cutoff) to remove the free MB remaining in solution. The supernatants were collected to analyze the concentration of the released MB. The concentration of MB in modules was calculated as follows: $c_{MB\ loading} = (n_{MB\ total} - n_{MB\ free})/V_{modules}$.

MiR-21-triggered DNA assembly

According to Table S2, differently valent TDNH1 and TDNH2 or h1 and h2 were mixed with miR-21. The mixture was then isothermally incubated at 37 °C for proper time.

Electrophoresis characterization

Agarose gel electrophoresis was applied to characterize the successful preparation of building modules and DNA assemblies. The reaction solutions were separated by 2% agarose gel electrophoresis at 4 °C (80 V, 30 min). The gels were stained with GelRed (Takara) in advance and visualized with a Gel Image System.

Atomic force microscopy imaging

In air scan. The modules and DNA assemblies (1 µM, 10 µL) were deposited onto a freshly cleaved mica surface and left to adsorb to the surface for 5 min, washed with 30 µL of water for more than 10 times and dried with compressed air. A MultiMode V8 AFM (Bruker) system was used to image the samples under ScanAsyst-Air mode.

FRET measurement

To achieve the *in vitro* FRET sensing of miR-21, different concentrations of miR-21 were added in 100 µL of Tris-HCl solution (20 mM Tris with 10 mM $MgCl_2$, pH = 7.4) containing 50 nM $qTDNH1^{Cy3}$ and 50 nM $qTDNH2^{Cy5}$ or 50 nM $h1^{Cy3}$ and 50 nM $h2^{Cy5}$ ($H1^{Cy3}$, $H2^{Cy5}$, $h1^{Cy3}$ or $h2^{Cy5}$ is 50 nM), followed by incubation for proper time at 37 °C. Then, the fluorescence spectrum of the mixture was collected from 550 to 800 nm with 540 nm excitation. All experiments were repeated at least three times. To investigate the kinetics of DNA assembly process, real-time monitoring of fluorescence intensity at 560 nm was performed after miR-21 addition. The *in vitro* FRET sensing of Let-7a was conducted by using the above-described process.

The FRET efficiency was evaluated using the equation of $E=F_A/F_{SUM}$[1-5], where E is FRET efficiency, F_A is the integral area of acceptor Cy5 (from 620 to 800 nm) in the fluorescence spectrum of DNA assembly, F_{SUM} is the total integral area of the whole spectrum of DNA assembly.

Biostability characterization of modules and DNA assemblies

To compare the abilities of nuclease resistance, 1 µM h1, 1 µM qTDNH1, HCR products formed by h1 and h2, hyperbranched HCR products formed by qTDNH1 and qTDNH2 were respectively incubated with 0.5 U/mL DNase I at room temperature for different time. Then, 20 µL of reaction solution was mixed with 4 µL of loading buffer (6×), followed by 2% agarose gel electrophoresis running at 80 V for about 30 min in 1×TAE buffer and analyzed by a fluorescence image scanner.

To investigate the stabilities of HCR products in mimic cellular physiological environment, hyperbranched HCR products formed by $qTDNH1^{Cy3}$ and $qTDNH2^{Cy5}$ (or $h1^{Cy3}$ and $h2^{Cy5}$) were incubated with 10% fetal bovine serum (FBS) at 37 °C, and time-dependent FRET signal changes were monitored for 36 h.

Cell culture and confocal fluorescence imaging

HeLa, MCF-7 and HL-7702 cells were obtained from the Cell Bank of Type Culture Collection of the Chinese Academy of Sciences (Shanghai, China) and cultured in DMEM (GIBCO) medium with 10% FBS and 1% penicillin-streptomycin (PS, 10000 IU penicillin and 10000 µg/mL streptomycin). Then the cells were seeded on a glass slide in a Petri dish and cultured overnight to reach an appropriate cell density. The fluorescent-labelled modules (*e.g.* $qTDNH1^{Cy3}$ and $qTDNH2^{Cy5}$ or $qTDNH3^{Cy3}$ and

qTDNH4^{Cy5}) were incubated with different cells in culture medium and 5% CO_2 at 37 °C for 4 h. The fluorescence images of cells were recorded by confocal laser scanning microscopy (CLSM).

Treating HeLa cells with estradiol: Estradiol was first dissolved in ethyl alcohol and diluted to 10 nM with fresh medium. After being incubated with 10 nM estradiol for 24 h, HeLa cells were further incubated with qTDNH1^{Cy3} and qTDNH2^{Cy5} for 4 h in culture medium and 5% CO_2 at 37 °C. Then, the fluorescence images of cells were recorded by CLSM.

Transfecting HeLa with DNA mimics of miR-21 and antimiR-21: Transfection assays were performed according to the manufacturer's protocol. Briefly, 10 µL of Lipofectamine 3000 reagent was firstly diluted with 190 µL of Opti-MEM (solution 1). The reaction solution (200 µL, called solution 2) was prepared by mixing 300 nM DNA mimics of miR-21 or antimiR-21 with opti-MEM. The final transfection liquid solution (400 µL) was obtained by mixing solution 1 with solution 2 and then kept in a static state for 15 min. The cells were incubated with the prepared transfection liquid solution for 4 h at 37 °C. Then, qTDNH1^{Cy3} and qTDNH2^{Cy5} were incubated with different cells in culture medium at 37 °C in 5 % CO_2 for 4 h, followed by imaging cells using CLSM. Transfection MCF cells with DNA mimics or inhibitors of Let-7a was conducted by using the above-described process.

$^1O_2$ detection

DPBF and DCFH-DA were used as $^1O_2$ probes to detect the generation of $^1O_2$ in solution and in cells, respectively. $^1O_2$-probing by DPBF was performed by mixing qTDNH1@MB in Tris buffer with 0.01 mg/mL DPBF in 10% ethanol, followed by photoirradiation with 650 nm laser (30 mW/cm^2). The absorbance values of DPBF at 410 nm were recorded using a UV–vis spectrophotometer. The solution of DPBF without qTDNH1@MB was used as the control.

Intracellular $^1O_2$-probing was performed by using DCFH-DA as the probe. Briefly, HeLa cells were placed in a 35 mm cell well for 24 h and grew to ~80% confluence. The culture medium was then replaced by a fresh medium containing qTDNH1@MB+qTDNH2@MB (Each qTDN: 12.5 nM, H1 or H2: 50 nM, MB: 80 nM) or free MB (80 nM). After incubation for 4 h, previous medium was changed as the well-prepared DCFH-DA staining solution (10 µM). The cells were incubated for another 30 min. Then, the cells were irradiated (650 nm, 30 mW/cm^2) for 3 min. All of the cells were observed by CLSM with a laser at 488 nm.

PDT treatment of cancer cells

HeLa cells were cultured in 96-well plates for 24 h and treated with different concentrations of qTDNH1^{Cy3}@MB+qTDNH2^{Cy5}@MB, qTDNH1^{Cy3}+qTDNH2^{Cy5}, or free MB for 4 h. After washing with fresh media, the cells were incubated for 24 h and subsequently treated with or without laser irradiation (650 nm and 30 mW/cm^2) for 3 min. The cell viability was then evaluated *via* MTT assay.

2. AGE and AFM characterization of TDN-assembled hairpins and their HCR products

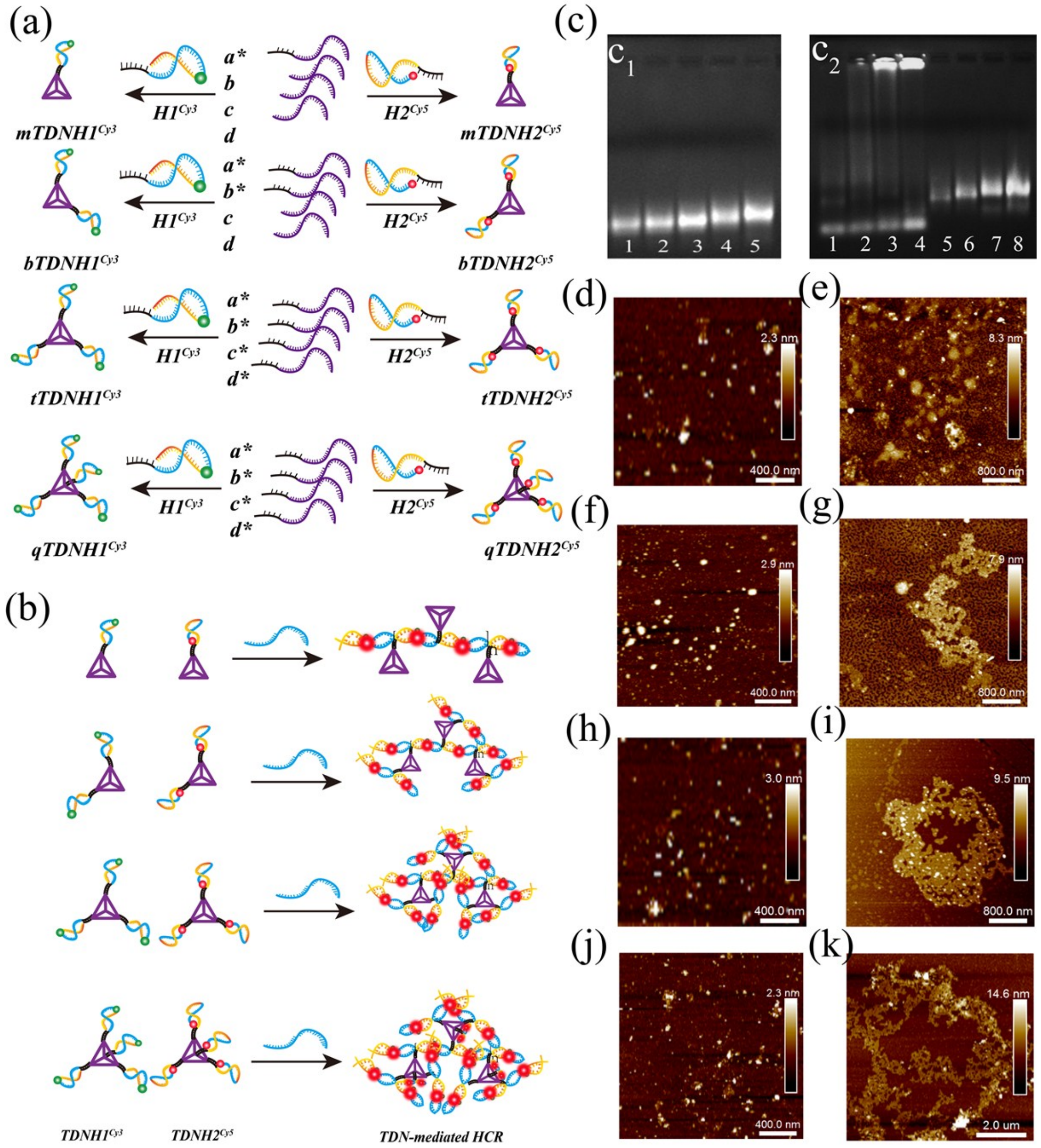

Fig. S1 Characterization of valence-controlled TDN-assembled hairpins and their HCR products. (a-b) Schematic illustration of TDN-assembled hairpins and their HCR products with different valences. (c) Gel images showing the successful preparation of TDN-assembled hairpins and the successful performance of corresponding HCR reactions. (c_1) Preparation of TDN-assembled hairpins with different valences. Lanes 1→5: TDN; mTDNH1; bTDNH1; tTDNH1; qTDNH1. (c_2) miR-21-triggered HCR reactions of TDN-assembled hairpins with different valences. Lanes 1→8: mTDNH1+mTDNH2+miR-21; bTDNH1+bTDNH2+miR-21; tTDNH1+tTDNH2+miR-21; qTDNH1+qTDNH2+miR-21; mTDNH1+mTDNH2; bTDNH1+bTDNH2; tTDNH1+tTDNH2; qTDNH1+qTDNH2. (d-k) AFM images of the morphology of (d) mTDNH1+mTDNH2, (e) mTDNH1+mTDNH2+miR-21, (f) bTDNH1+bTDNH2, (g) bTDNH1+bTDNH2+miR-21, (h) tTDNH1+tTDNH2, (i) tTDNH1+tTDNH2+miR-21, (j) qTDNH1+qTDNH2 and (k) qTDNH1+qTDNH2+miR-21.

3. **Reaction kinetics and FRET efficiency of different valence TDN-mediated HCR**

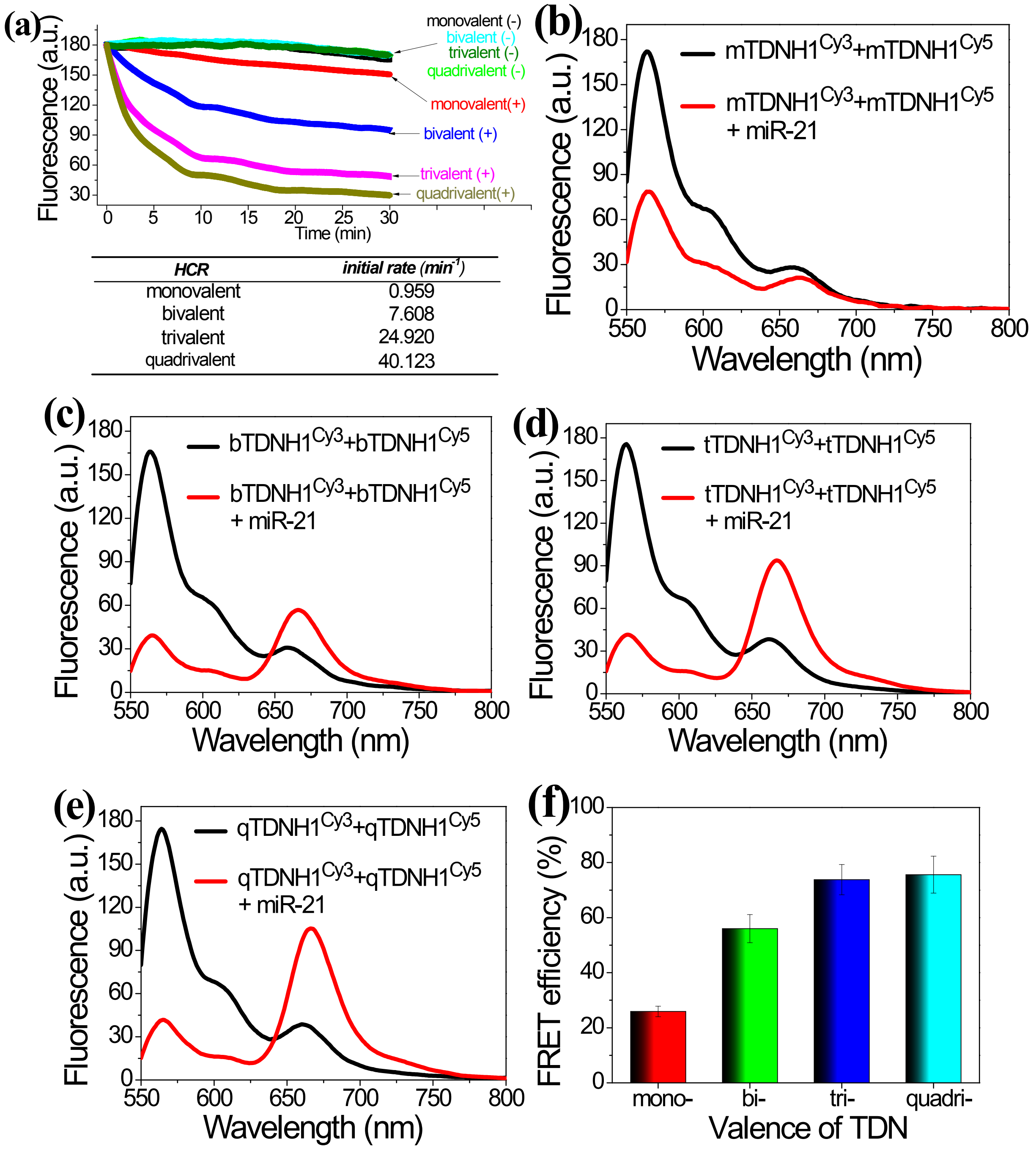

HCR	*initial rate (min^{-1})*
monovalent	0.959
bivalent	7.608
trivalent	24.920
quadrivalent	40.123

Fig. S2 (a) Time-dependent Cy3 fluorescence changes (at 560 nm) of different valence TDN-mediated HCR in the absence (-) or presence (+) of 20 nM miR-21. The initial rates of TDN-mediated HCR were outlined in the table below. (b-e) Fluorescence spectra of different valence TDN-mediated HCR reaction systems reaching equilibriumin the absence or presence of 20 nM miR-21. λ_{ex} = 540 nm. (f) FRET efficiencies calculated from above four HCR systems. [$H1^{Cy3}$] = [$H2^{Cy5}$] = 50 nM. The concentrations of TDN in mTDNH1, bTDNH1, tTDNH1 and qTDNH1 were 50 nM, 25 nM, 16.67 nM and 12.5 nM, respectively.

4. FRET images of miR-21 in HeLa cells at different time points

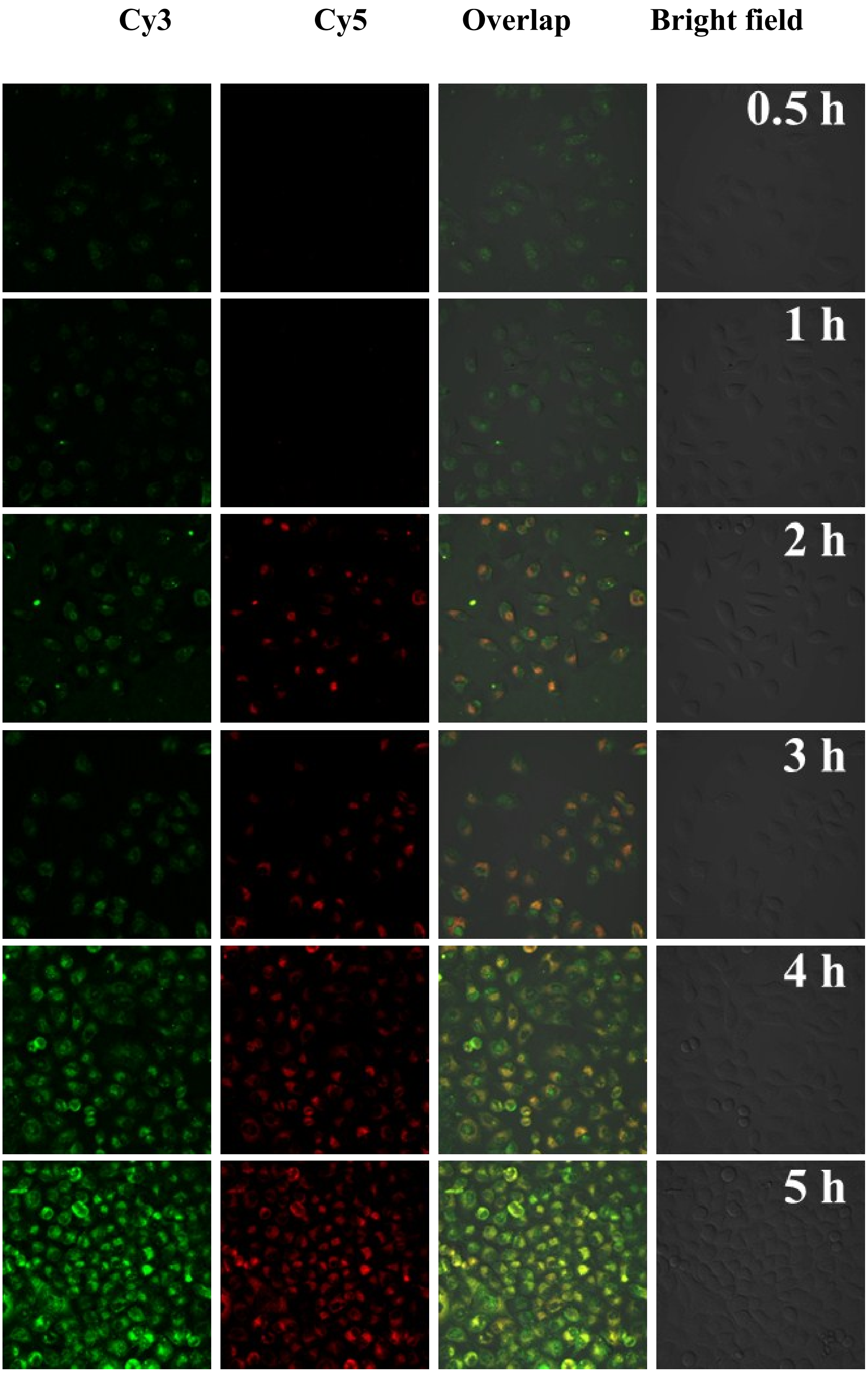

Fig. S3 FRET images of miR-21 in HeLa cells recorded at different time points. Herein, qTDNH1^{Cy3} and qTDNH2^{Cy5} were used. The concentrations of hairpins of H1^{Cy3} and H2^{Cy5} are all 50 nM. qTDN in qTDNH1^{Cy3} or qTDNH2^{Cy5} was 12.5 nM in intracellular target imaging systems.

5. FRET measurement of Let-7a in solutions and MCF-7 cells

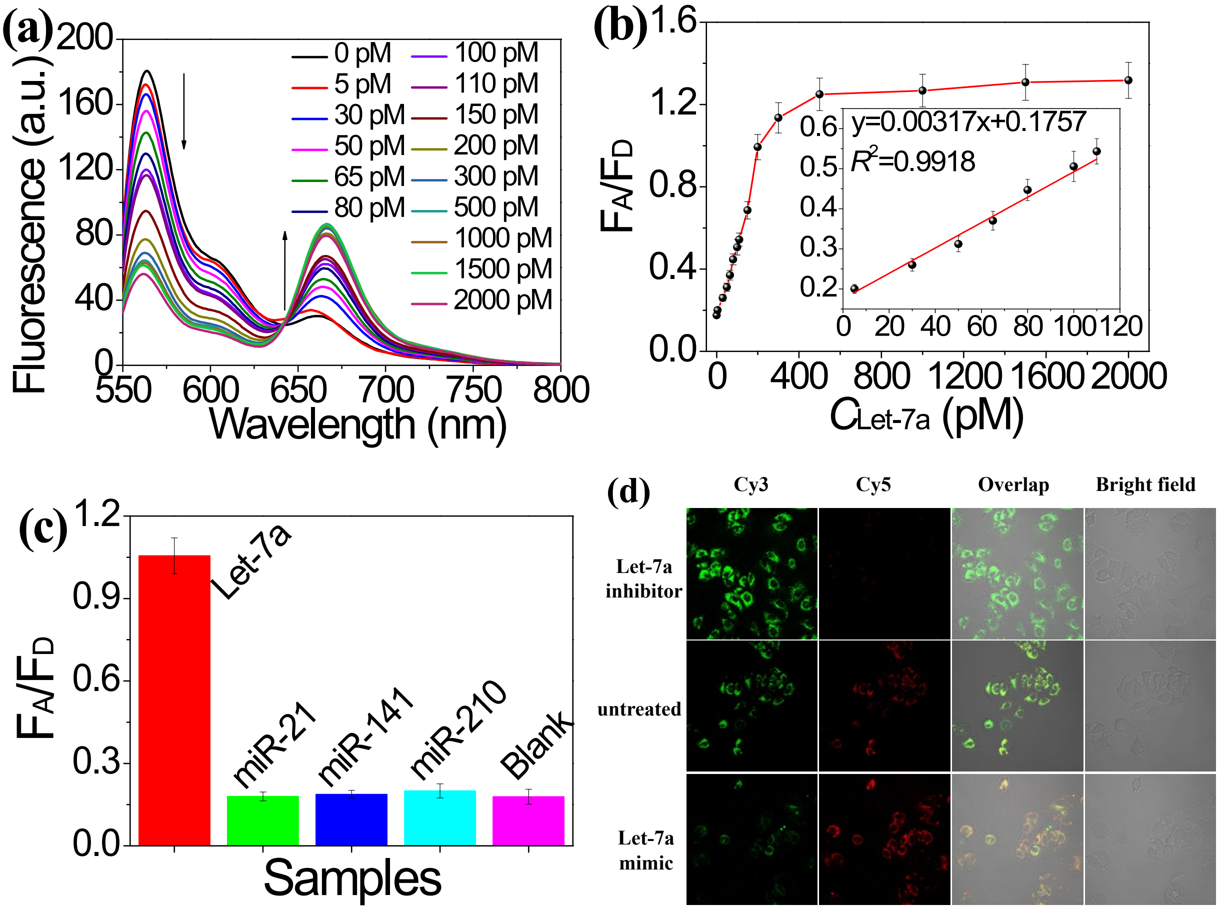

Fig. S4 Let-7a-specific sensing and in situ imaging using qTDN-mediated hyperbranched HCR. (a) Fluoresence spectral change of the sensing system in the presence of different concentrations of Let-7a. (b) Let-7a concentration-dependent changes in F_A/F_D and the linear relationship (inset) between F_A/F_D and Let-7a concentration in the range of 5 pM-110 pM. (c) Selectivity of qTDN-mediated hyperbranched HCR-based Let-7a-sensing system. 200 pM of Let-7a or 20 nM of other different microRNAs were added. (d) FRET images of Let-7a in untreated, Let-7a mimic or inhibitor-transfected MCF-7 cells. The concentrations of hairpins of $H3^{Cy3}$ and $H4^{Cy5}$ were 50 nM and qTDN in $qTDNH3^{Cy3}$ or $qTDNH4^{Cy5}$ was 12.5 nM.

6. Cytotoxicities of hairpins and qTDN-assembled hairpins

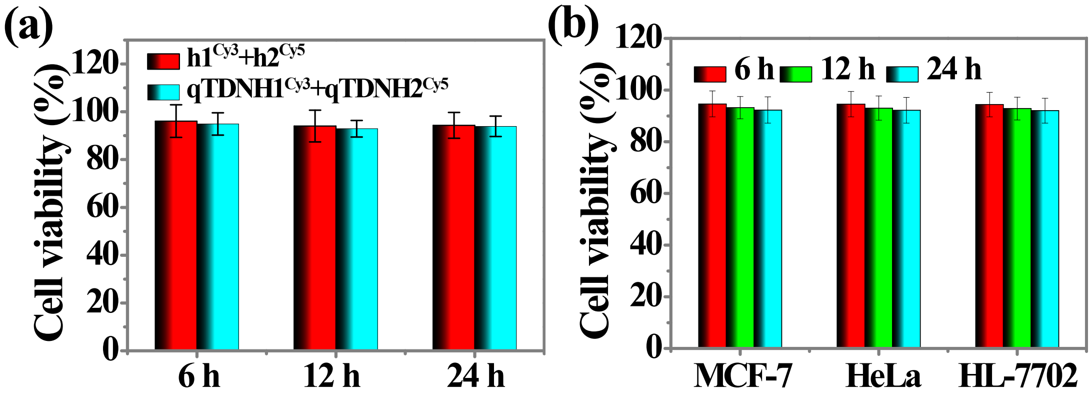

Fig. S5 MTT assay of hairpins and qTDN-assembled hairpins. (a) Cell viabilities of HeLa cells treated with $h1^{Cy3}$+$h2^{Cy5}$ or $qTDNH1^{Cy3}$+$qTDNH2^{Cy5}$ for different time. (b) Cell viabilities of different cells treated with $qTDNH1^{Cy3}$ plus $qTDNH2^{Cy5}$ for different time. The concentrations of hairpins of $h1^{Cy3}$, $h2^{Cy5}$, $H1^{Cy3}$ and $H2^{Cy5}$ are all 50 nM. qTDN in $qTDNH1^{Cy3}$ or $qTDNH2^{Cy5}$ was 12.5 nM.

7. Effects of MB loading on FRET-based miR-21-sensing

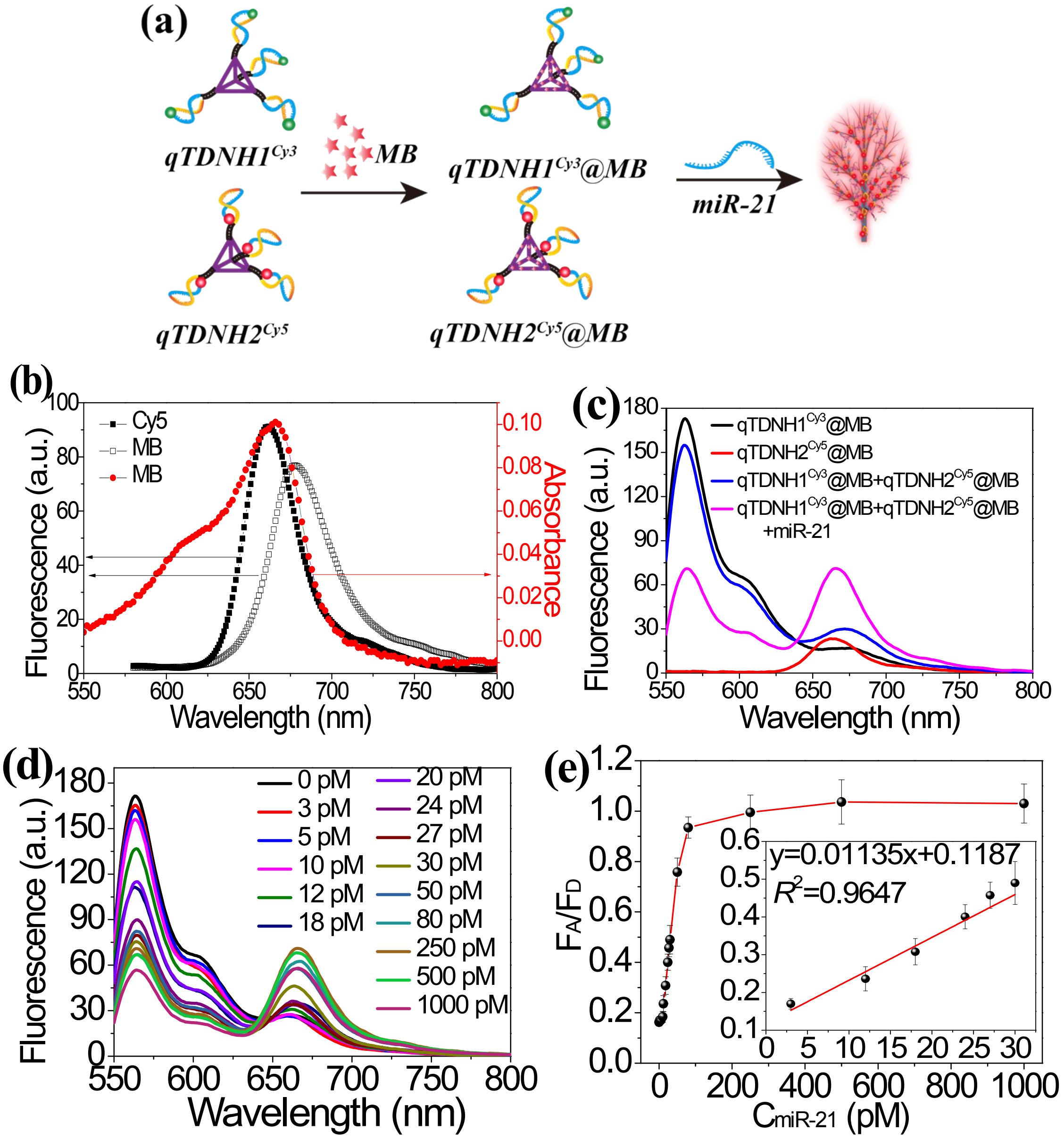

Fig. S6 qTDN-mediated hyperbranched HCR-based miR-21-sensing in the presence of MB. (a) Working mechanism. (b) UV-vis absorption spectrum of MB and fluorescence spectra of Cy5 and MB. (c) Fluorescence spectra given by the systems containing qTDNH1^{Cy3}@MB, qTDNH2^{Cy5}@MB, qTDNH1^{Cy3}@MB+qTDNH2^{Cy5}@MB or qTDNH1^{Cy3}@MB+qTDNH2^{Cy5}@MB+miR-21. [miR-21] = 20 nM. λ_{ex} = 540 nm. (d) miR-21 concentration-dependent changes in fluorescence spectra; (e) miR-21 concentration-dependent changes in F_A/F_D signal (F_A and F_D are the fluorescence of Cy5 at 660 nm and Cy3 at 560 nm, respectively). The inset figure shows the linear relationship between F_A/F_D and the miR-21 concentration in the range of 3 pM-30 pM.

8. Comparation of our microRNA-sensing system with reported ones

Table S3. Comparison of several RNA detection methods

Method	Target	Detection limit	Ref
qTDN-mediated HCR	microRNA	2.14 pM	this work
Protein scaffolded DNA tetrads	microRNA	6 pM	(6)
Nucleic acid molecular aggregates	microRNA	10 pM	(7)
DNAzyme based amplification	microRNA	100 pM	(8)
Au@PDA NPs	microRNA	10 nM	(9)
ATP-fueled DNA nanomachine	microRNA	100 pM	(10)
FRET based DNA tetrahedron nanotweezer	mRNA	330 pM	(11)

References

1. A. Giri, A. Makhal, B. Ghosh, A. K. Raychaudhuri and S. K. Pal, *Nanoscale*, 2010, **2**, 2704-2709.
2. Z. Li, Y. Wang, G. Zhang, W. Xu and Y. Han, *J. Lumin.*, 2010, **130**, 995-999.
3. J. Wlodarczyk, A. Woehler, F. Kobe, E. Ponimaskin, A. Zeug and E. Neher, *Biophys. J.*, 2008, **94**, 986-1000.
4. B. Vasudevanpillai, I. Tamitake, B. Yoshinobu and I. Mitsuru, *J. Phys. Chem. B.*, 2006, **110**, 26068-26074.
5. H. Q. Wang, Y. Q. Li, J. H. Wang, X. Qiao, X. Q. Li and Y. D. Zhao, *Anal. Chim. Acta.*, 2008, **610**, 68-73.
6. D.-J. Huang, Z.-M. Huang, H.-Y. Xiao, Z.-K. Wu, L.-J. Tang and J.-H. Jiang, *Chem. Sci.*, 2018, **9**, 4892-4897.
7. Z. Zhang, Y. Wang, N. Zhang and S. Zhang, *Chem. Sci.*, 2016, **7**, 4184-4189.
8. J. Liu, M. Cui, H. Zhou and W. Yang, *ACS Sensors*, 2017, **2**, 1847-1853.
9. C. K. K. Choi, J. Li, K. Wei, Y. J. Xu, L. W. C. Ho, M. Zhu, K. K. W. To, C. H. J. Choi and L. Bian, *J. Am. Chem. Soc.*, 2015, **137**, 7337-7346.
10. P.-Q. Ma, C.-P. Liang, H.-H. Zhang, B.-C. Yin and B.-C. Ye, *Chem. Sci.*, 2018, **9**, 3299-3304.
11. L. He, D. Q. Lu, H. Liang, S. Xie, C. Luo, M. Hu, L. Xu, X. Zhang and W. Tan, *ACS Nano*, 2017, **11**, 4060-4066.
